# Protective effect of yerba mate intake on the cardiovascular system: a *post hoc* analysis study in postmenopausal women

**DOI:** 10.1590/1414-431X20187253

**Published:** 2018-04-19

**Authors:** D.T.A. da Veiga, R. Bringhenti, R. Copes, E. Tatsch, R.N. Moresco, F.V. Comim, M.O. Premaor

**Affiliations:** 1 Departamento de Clínica Médica, Centro de Ciências da Saúde, Universidade Federal de Santa Maria, Santa Maria, RS, Brasil; 2 Laboratório de Bioquímica Clínica, Departamento de Análises Clínicas e Toxicológicas, Universidade Federal de Santa Maria, Santa Maria, RS, Brasil

**Keywords:** Yerba mate, Ilex paraguariensis, Postmenopausal women, Diabetes mellitus, Hypertension, Cardiovascular disease

## Abstract

The prevalence of cardiovascular and metabolic diseases is increased in postmenopausal women, which contributes to the burden of illnesses in this period of life. Yerba mate (*Ilex paraguariensis*) is a native bush from Southern South America. Its leaves are rich in phenolic components, which may have antioxidant, vasodilating, hypocholesterolemic, and hypoglycemic proprieties. This *post hoc* analysis of the case-control study nested in the Obesity and Bone Fracture Cohort evaluated the consumption of yerba mate and the prevalence of hypertension, dyslipidemia, and coronary diseases in postmenopausal women. Ninety-five postmenopausal women were included in this analysis. A questionnaire was applied to evaluate the risk factors and diagnosis of cardiovascular diseases and consumption of yerba mate infusion. Student's *t*-test and chi-square test were used to assess significant differences between groups. The group that consumed more than 1 L/day of mate infusion had significantly fewer diagnoses of coronary disease, dyslipidemia, and hypertension (P<0.049, P<0.048, and P<0.016, respectively). Furthermore, the serum levels of glucose were lower in the group with a higher consumption of yerba mate infusion (P<0.013). The serum levels of total cholesterol, LDL-cholesterol, HDL-cholesterol, and triglycerides were similar between the groups. This pragmatic study points out the benefits of yerba mate consumption for the cardiovascular and metabolic systems. The ingestion of more than 1 L/day of mate infusion was associated with fewer self-reported cardiovascular diseases and lower serum levels of glucose. Longitudinal studies are needed to evaluate the association between yerba mate infusion and reduction of cardiovascular diseases in postmenopausal women.

## Introduction

Menopause is associated with an increased frequency of chronic conditions, such as hypertension, cardiovascular disease (CVD), metabolic syndrome, and diabetes mellitus (DM). Traditional cardiovascular risk factors are usually more prevalent after menopause because of loss of estrogenic protection. Menopause has been associated with dyslipidemia, hyperglycemia, and hypertension. The prevalence of hypertension in postmenopausal women is two times higher than in premenopausal women and more than 75% of women older than 60 years have hypertension (1). Moreover, type 2 DM is a disease associated with specific changes in the lipid profile promoting an atherogenic pattern (2). These findings contribute to the morbidity of CVD in postmenopausal women.

Yerba mate (*Ilex paraguariensis*) is a native plant from southern South America, consumed daily by ∼70% of that population in the form of mate, mate tea or *tererê*. Several classes of phytochemicals are found in its leaf composition: xanthines (such as caffeine and theobromine), saponins (derived from ursolic and oleanolic acids), and phenolic components (such as chlorogenic acid and other classes of polyphenols). Many experimental studies indicate antioxidant, anti-inflammatory, vasodilating, hypocholesterolemic, and hypoglycemic properties related to yerba mate use (3). In humans, some experimental studies have pointed out a beneficial effect of the consumption of yerba mate on the levels of total cholesterol (CT), triglycerides (TG), and glucose (4–6). The aim of this study was to evaluate the association between the consumption of yerba mate and the frequencies of dyslipidemia, DM, and CVDs in postmenopausal women.

## Material and Methods

This study was a *post hoc* analysis of the case-control study (7) nested in the Obesity and Bone Fracture Cohort (8) that aimed to evaluate the association between bone fracture and the intake of the aqueous extract of mate tea consumed as “*chimarrão*” in postmenopausal women. It was conducted in the municipality of Santa Maria, RS, Brazil. The original cohort recruited 1057 women aged >55 years at primary care facilities. Women at menacme and those with cognitive deficits or communication difficulties were excluded from the survey. All 46 women with confirmed major bone fractures in the original cohort and 49 randomly selected controls without fractures (7) were included in this *post hoc* analysis (n=95).

Approval for this study was obtained from the Ethics Committee of the Universidade Federal de Santa Maria (CAAE 04320312.2.0000.5346) and from the Secretaria de Saúde da Prefeitura de Santa Maria (Ofício 492/2012/SMS/NEPeS) and followed the Declaration of Helsinki principles. All participants who agreed to participate in the study signed the informed consent term.

A standardized questionnaire including risk factors for CVD and self-reported CVD was applied at baseline (8–10). Yerba mate intake was evaluated by a food frequency questionnaire. The intake of the aqueous extract of yerba mate consumed as “*chimarrão*” (mate) was quantified per day. More information regarding data collection is shown in the Supplementary Material.

Serum albumin, CT, LDL-cholesterol (LDL-C), HDL-cholesterol (HDL-C), TG, glucose, and creatinine were measured using the standard methods on Cobas MIRA¯ (Roche Diagnostic, Switzerland) automated analyzer. The serum levels of nitrite/nitrate (NO), ferric reducing ability of plasma (FRAP), and advanced oxidation protein products (AOPP) also were measured using Cobas MIRA¯.

Statistical analysis was performed using the IBM SPSS program (version 19.0 for Windows, Brazil). Data are reported as prevalence rates (percentages) or means (standard deviations). The cutoff considered for mate consumption was 1 L/day, a criterion adapted and modified from Conforti et al. (11). The chi-square and Student's *t*-tests were used to determine possible differences between the two groups. Differences were considered significant when the two-tailed P*-*value was less than 0.05. The power of this study for the evaluation of the association between mate intake and the frequency of coronary disease was 0.991.

## Results

The characteristics of the original cohort and the case-control study are described in detail elsewhere (7,8). Table 1 shows the clinical characteristics of the participants with a daily consumption of mate. There was no difference between groups regarding age, body mass index, waist circumference, physical activity, tobacco use, and years of schooling. The women who drank at least 1 L/day of mate had significantly fewer diagnoses of dyslipidemia, hypertension, and coronary disease (Table 1).

**Table 1.** Clinical characteristics of the studied women according to their daily intake of yerba mate.

Characteristics	<1 L of MATE^a^ (n=78)	≥1 L of MATE^b^ (n=17)	P-value
Age (years)	69.2±6.6	68.8±9.8	0.847^c^
BMI (kg/m^2^)	29.4±6.2	29.4±5.0	0.992^c^
Waist circumference (cm)	97.5±11.0	100.8±11.0	0.299^c^
Diastolic blood pressure (mmHg)	80.4±13.1	82.4±8.6	0.435^c^
Systolic blood pressure (mmHg)	139.5±23.0	136.5±26.9	0.672^c^
Physical activity (Baecke's score)	7.2±1.5	6.9±1.1	0.486^c^
Tobacco use	12.8	5.9	0.418^d^
Health insurance	55.8	64.3	0.504^d^
Years of schooling (≤8 years)	65.3	68.8	0.794^d^
Diagnoses			
Dyslipidemia	61.5	35.3	0.048^d^
Hypertension	66.7	35.3	0.016^d^
Diabetes mellitus	23.1	11.8	0.300^d^
Metabolic syndrome	66.6	52.9	0.412^d^
Stroke	9.0	0	0.199^d^
Coronary disease	19.2	0	0.049^d^
Heart failure	2.6	5.9	0.478^d^

Data are reported as means±SD or percentage. ^a^Women who did not drink mate infusion and those who drank less than 1 L/day. ^b^Women who had a daily consumption of mate infusion at or above 1 L. ^c^Student's *t*-test. ^d^Chi-square test.

There was no significant difference in the levels of albumin, serum lipids, markers of oxidative stress, and creatinine between the women with mate intake equal to or greater than 1 L/day and women with a lesser intake. The fasting serum levels of glucose were lower in the women with a higher consumption of mate (Table 2).

**Table 2.** Laboratory characteristics of the studied women according to their daily intake of yerba mate.

Characteristics	<1 L of MATE^a^ (n=78)	≥1 L of MATE^b^ (n=17)	P-value^c^
Albumin (g/dL)	4.48 (0.84)	4.30 (0.82)	0.579
Total cholesterol (mg/dL)	112.3 (33.0)	99.0 (14.0)	0.917
LDL (mg/dL)	130.9 (50.9)	121.4 (42.2)	0.917
HDL (mg/dL)	54.6 (16.0)	53.0 (13.9)	0.709
Triglyceride (mg/dL)	168.6 (79.0)	182.4 (123.0)	0.481
Glucose (mg/dL)	112.3 (33.0)	99.0 (14.0)	0.013
Creatinine (mg/dL)	0.73 (0.20)	0.63 (0.16)	0.062
Ln (NOx)	5.04 (0.89)	5.20 (0.89)	0.557
Ln (FRAP)	6.07 (0.51)	5.90 (0.72)	0.299
Ln (AOPP)	3.35 (0.77)	3.29 (0.90)	0.807

Data are reported as means±SD. ^a^Women who did not drink mate infusion and those who drank less than 1 L/day. ^b^Women who had a daily consumption of mate infusion at or above 1 L. ^c^Student's *t*-test. NOx: levels of nitrite/nitrate; FRAP: ferric reducing ability of plasma; AOPP: advanced oxidation protein products. Ln: natural logarithm.

## Discussion

In this *post hoc* analysis, postmenopausal women who consumed more than 1 L/day of mate reported fewer diagnoses of dyslipidemia, hypertension, and coronary disease. Although some metabolic findings have been reported in previous studies (4–6,12), to the best of our knowledge, this is the first study to report the beneficial effect of yerba mate consumption on the incidence of chronic diseases in postmenopausal women.

The acute effect of mate tea on blood pressure was first described by de Morais et al. They evaluated the impact of an intake of 1 L/day of mate tea within 40 days in adult subjects and found a reduction of 2.3% in systolic blood pressure in the experimental group (4). Although we did not find an effect on the blood pressure measured at the time of blood sample collection, the women in our study presented significantly fewer diagnoses of hypertension and coronary disease. However, the exact mechanism by which yerba mate consumption has favorable effects on the cardiovascular system is not known. Schinella et al. (13) described the protective effect of the aqueous extract of mate on myocardial dysfunction induced by ischemia and reperfusion in an experimental animal model. In their study, the cardioprotective effect appears to be linked to an NO-dependent mechanism (13). Other studies conducted on rats fed a high-fat diet found that mate aqueous extract showed beneficial effects on the vascular endothelium (14,15).

In our study, the women with a higher consumption of mate had fewer diagnoses of dyslipidemia, despite the fact that no significant differences were observed in the evaluation of the serum levels of CT, LDL-C, HDL-C, and TG. These results might suggest that mate intake could have a long-term effect or an indirect mechanism. Different studies have reported different effects on the serum lipid levels (4,5,16). In a study conducted on adult subjects, the intake of 990 mL/day of mate for 40 days decreased the serum levels of CT and LDL-C and increased the serum levels of HDL-C in subjects with dyslipidemia. Conversely, only the serum levels of LDL-C decreased in normolipemic individuals (4). These findings suggest that yerba mate consumption might have a different effect on lipid levels depending on the previous metabolic status of the subject. Another study also conducted in middle-aged subjects described a decrease in the serum levels of TC and LDL-C but no changes in the serum levels of TG (5).

In the present study, a significant difference was observed in serum glucose levels between the higher-consumption mate group and the controls. The reduction in serum glucose levels and improvement in insulin resistance have been described in previous studies (6,16). In a clinical trial, Boaventura et al. (16) evaluated the consumption of 1 L of mate per day for 60 days on glycemia, advanced glycation end-products (HbA1c), and oxidative stress in subjects with DM and pre-DM. They observed reductions in the levels of glucose, HbA1c, and lipid peroxidation, suggesting that yerba mate intake could have a long-term beneficial effect on DM.

In the postmenopausal period and consequent estrogen reduction, there is an imbalance in oxidative stress, with decreased antioxidant defenses and increased oxidation, mainly lipid peroxidation (17). In our study, the markers of oxidative stress were not modified by the consumption of mate, which was partly different from the findings of the study by Boaventura et al. (16). Although they found no change in serum levels of FRAP, there was an increase in the erythrocyte levels of reduced glutathione after 60 days of treatment with 1 L of mate per day.

Several factors may explain the negative findings of our study regarding oxidative stress. First, the markers in our study were measured from samples of blood instead of liver. Sometimes, serum markers may not reflect what is occurring in other tissues. Second, many variables other than yerba mate intake in non-experimental conditions, such as the systemic inflammation present in CVDs, could influence the markers of oxidative stress. Finally, the preparation and consumption manner of yerba mate may also have affected the results (18,19).

The main strength of our study is its pragmatic nature with a sample representative of the Brazilian primary care users, which makes the generalization of the findings more intuitive. However, it also has some weaknesses. The study is a *post hoc* analysis; therefore, the results must be confirmed with other studies. The small sample size could have contributed to the negative results in blood pressure, blood lipid levels, and oxidative stress. Blood pressure was only measured once at the time of blood sample collection. We also did not have information on the patients' treatment, which may have affected blood pressure and biochemistry measurements. Moreover, the diagnoses were self-reported. Nonetheless, there is no reason to believe that the recall bias was different between mate drinkers and non-mate drinkers.

In conclusion, we described a potential beneficial effect of yerba mate use on the cardiovascular system, as indicated by fewer diagnoses of dyslipidemia, hypertension, and coronary disease in postmenopausal women.

## Supplementary Material

Click here to view [pdf].
